# Regulation of biological processes by ubiquitin ligases: a focus on the Pagano Lab’s contribution

**DOI:** 10.3389/fcell.2024.1458895

**Published:** 2024-08-15

**Authors:** Philipp Kaldis, Lisa A. Porter

**Affiliations:** ^1^ Department of Clinical Sciences, Lund University, Clinical Research Centre (CRC), Malmo, Sweden; ^2^ Lund University Diabetes Centre (LUDC), Lund University, Clinical Research Centre (CRC), Malmo, Sweden; ^3^ Department of Biomedical Sciences, University of Windsor, WE-SPARK Health Institute, Windsor, ON, Canada; ^4^ Lawson Research Institute, St. Joseph’s Healthcare London, London, ON, Canada

**Keywords:** ubiquitin ligase, ubiquitin 26S-proteasome system, protein degradation, cell cycle regulation, ubiquitin

## Abstract

Protein homeostasis depends on many fundamental processes including mRNA synthesis, translation, post-translational modifications, and proteolysis. In the late 70s and early 80s the discovery that the small 76 amino acid protein ubiquitin could be attached to target proteins via a multi-stage process involving ubiquitin-activating enzymes, ubiquitin conjugating enzymes, and ubiquitin ligases, revealed an exciting new post-translational mechanism to regulate protein degradation. This cellular system was uncovered using biochemical methods by Avram Hershko, who would later won the Nobel prize for this discovery; however, the biological functions of ubiquitin ligases remained unknown for many years. It was initially described that ubiquitin modifies proteins at one or more lysine residues and once a long ubiquitin chain was assembled, proteins were degraded by the proteasome. Now we know that proteins can be mono-, multimono-, homotypic poly-, or heterotypic poly-ubiquitylated, each of which confers a specific signal that goes beyond protein degradation regulating additional key cellular functions such as signal transduction, protein localization, recognition of damaged proteins, etc.

## Introduction

Protein homeostasis depends on many fundamental processes including mRNA synthesis, translation, post-translational modifications, and proteolysis. In the late 70s and early 80s the discovery that the small 76 amino acid protein ubiquitin could be attached to target proteins via a multi-stage process involving ubiquitin-activating enzymes, ubiquitin conjugating enzymes, and ubiquitin ligases, revealed an exciting new post-translational mechanism to regulate protein degradation. This cellular system was uncovered using biochemical methods by Avram Hershko, who would later won the Nobel prize for this discovery (Varshavsky, 2006); however, the biological functions of ubiquitin ligases remained unknown for many years. It was initially described that ubiquitin modifies proteins at one or more lysine residues and once a long ubiquitin chain was assembled, proteins were degraded by the proteasome. Now we know that proteins can be mono-, multimono-, homotypic poly-, or heterotypic poly-ubiquitylated, each of which confers a specific signal that goes beyond protein degradation regulating additional key cellular functions such as signal transduction, protein localization, recognition of damaged proteins, etc. [for review see (Dikic and Schulman, 2023)].

Cell cycle progression is a highly regulated process that ensures accurate replication of the genetic material of a cell prior to distribution to two daughter cells. Integrity of the process depends on the regulated production and destruction of proteins known as cyclins, which in turn activate cyclin-dependent kinases (CDKs). In the mid 90s, elegant genetic work in yeast, as well as biochemical studies in higher eukaryotes suggested that cyclins were degraded via the ubiquitin system. In the meantime, researchers realized that there were several hundred ubiquitin ligases encoded in the human genome, starting a race to determine their functions. It became clear that the functions of ubiquitin ligases extend way beyond the cell cycle and seem to be involved in many biological processes. The laboratory directed by Michele Pagano contributed substantially to these studies and, therefore, we decided to sit down with him for a “Fireside Chat.” This conversation reveals the motivations and journey of Dr. Pagano, one of the top cancer biologists performing mechanistic studies in the fields of cell cycle control and ubiquitin-mediated degradation. We aim to highlight Dr. Pagano’s scientific success from his viewpoint and share his thoughts on where the field is heading, as well as potential challenges and opportunities.

## Contributions by Michele Pagano and his laboratory

Dr. Pagano obtained an MD and a “specialty diploma” degree (similar to a PhD) in molecular endocrinology from the University of Naples Federico II School of Medicine, Italy. He then did a postdoc with Giulio Draetta at the EMBL in Heidelberg, Germany, and, after Draetta’s move, at the biotech company Mitotix in Boston. Next, he became an Assistant Professor at the New York University School of Medicine (NYUSoM) where he rose thru the ranks and since 2015 has served as the Chair of the Department of Biochemistry and Molecular Pharmacology. His research has been supported by the NIH continuously since 1998 and by the Howard Hughes Medical Institute (HHMI) since 2008.

### What made you decide to become a scientist? You started out as a physician. Was there a specific event that led you to discovery-based research?

My interest in science began in my native Naples during high school thanks to the influence of Ugo Moncharmont, a professor of biology. Prof. Moncharmont encouraged me to do an internship in a laboratory located in the basement of the school, culturing monocellular eukaryotic cells, mostly paramecia, and dissecting various species of fish, including little sharks. The great enthusiasm with which Prof. Moncharmont taught biochemistry and biology classes was contagious. He had also a consulting position at the Stazione Zoologica Anton Dohrn, an aquarium and an outstanding research center that attracted scientists from all over the world. Moncharmont often brought some of us to visit the laboratories of the aquarium. Because of these experiences, I wanted to go into basic research in biology, but my father, an MD, hoped that I too would get a medical degree. So, he convinced me to attend medical school with the idea that I could decide later between working as a clinician (as he wanted) or as a researcher (as I wanted). What he forgot is that, during the first 2 years of medical school, which in Italy starts immediately after high school, they teach hard core basic sciences (physics, chemistry, biochemistry, and biology) and this cemented my passion even more. During the last 2 years of my MD degree, I did an internship in general pathology and then, upon graduation I stayed on as a molecular endocrinology fellow to obtain my specialty diploma degree. During these years, I learned the basics of experimental biochemistry and cell biology investigating the regulation of the estrogen receptor.

### You completed a successful postdoctoral fellowship – can you give us context on how you decided to join the Draetta lab and what this experience was like?

I reached out to Giulio Draetta, who, after a very successful postdoctoral experience with David Beach at the Cold Spring Harbor Laboratory, had become an independent PI in the same research institute. Giulio accepted me as a postdoc around the same time as he accepted a new position at EMBL, hence instead of moving to New York, I moved to Heidelberg, Germany, for my postdoc. For 2.5 years I had a great experience, surrounded by the international environment of the EMBL and the rich collaborations that we were able to engage there. I was lucky enough to publish three papers in a short time (Pagano et al., 1992a; Pagano et al., 1992b; Pagano et al., 1992c). The first two through a collaboration with Dr. Pidder Jansen-Dürr at the Deutsches Krebsforschungszentrum, also in Heidelberg. These studies were focused on my interest in cyclins, activators of CDKs and how these proteins function to promote DNA replication during S-phase. While all of this was going on, Giulio decided to start a biotech company, Mitotix, which I co-founded. We moved to Cambridge, United States, to set up the company where I started to work on a project aimed at understanding why cyclin D1 is degraded prior to S-phase. We discovered that cells depleted of cyclin D1 synthesize more DNA during DNA repair, suggesting that cyclin D1 inhibits long resection upon DNA damage (Pagano et al., 1994). However, it was not until 30 years later (!) that we would understand the function of cyclin D1 in DNA repair, with us demonstrating that cyclin D1 keeps the mismatch repair pathway in check during G1 (Rona et al., 2024). At Mitotix, I also took on a different project that led to the discovery that p27^Kip1^, an inhibitor of CDKs, is degraded by the ubiquitin system. This was really novel and well received as, at the time, p27 became one of only two examples of cell cycle regulators being degraded by the ubiquitin system. We published this study in 1995 (Pagano et al., 1995) and after that, I decided to go back to academia and follow my interest in basic research. I applied for an assistant professorship at NYUSoM, where I continue to work today.

### What influenced you to select NYUSoM as an academic institution?

I had offers from three institutions in New York City and I am not completely sure why I chose NYUSoM. Partially, it was the respect for Vittorio DeFendi, the person who recruited me–among all the Italian scientists I have ever met, he was one of the warmest and most cultured individuals.

### Earlier, you mentioned the importance of collaborations; how have collaborations shaped and impacted your career?

My successes have been heavily influenced by collaborations. Among the most notable was the one with Avram Hershko, who I met at a conference in 1997. We discussed working together to dissect p27 degradation – Avram was very keen to discover the role of ubiquitin in a physiological context. We agreed that he would spend a summer sabbatical in my lab, which actually became seven summer sabbaticals. Avram is also a very warm, authentic person. Even after winning the Nobel Prize for discovery of the ubiquitin system, he has remained not only a great scientist, but also a wonderful person. Having Avram in the lab early in my career not only impacted my science, but it was an amazing experience for everyone in the lab. We ended up publishing nine papers together, which is a great testimony of our collaboration.

### What are some significant changes in the focus of your lab over the years?

With Avram, we discovered that p27 is ubiquitylated via SKP2 (Carrano et al., 1999), which is a member of the family of F-box proteins, substrate receptors of CUL1-RING ubiquitin ligase (CRL1) complexes. Following the early focus on p27, my lab became interested in dissecting the functions of other F-box proteins, thinking that other members may also control the cell cycle. In fact, we discovered that β-TrCP targets upstream CDK regulatory proteins, such as CDC25A (Busino et al., 2003), EMI1 (Guardavaccaro et al., 2003), claspin (Peschiaroli et al., 2006), REST (Guardavaccaro et al., 2008), etc. Subsequently, we characterized the role of other F-box proteins (FBXO1, FBXO5, and FBXO11), as well as of substrate receptors of other CRLs in controlling the progression through the cell cycle.

To take an unbiased approach, we performed an siRNA screen to downregulate all 69 F-box proteins in humans and, using various phenotypes as readouts, we asked which of them played a role in cell cycle control. These types of screens can take your research in completely different directions; for example, through this screen we focused on certain F-box proteins only to realize that they are involved in processes outside of (or perhaps upstream to) the cell cycle, like cell signaling, transcription, translation, organelle biogenesis, and even the circadian clock. One notable example was FBXL3, whose downregulation resulted in the inhibition of the intra S-phase checkpoint. Yet, when we purified the FBXL3 complex, we did not find checkpoint regulatory proteins, but the two cryptochrome proteins (CRY1 and CRY2), key regulators of the circadian clock.

Admittedly, I was hesitant to explore the circadian clock at first; it was not until a postdoc, Luca Busino (now an Associate Professor at the University of Pennsylvania) convinced me that maybe the circadian clock controls the cell cycle. This could be one of the reasons for which the cell cycle of proliferating mammalian cells is 24 h. We still do not know how cryptochrome proteins regulate the cell cycle, but Luca’s study turned into a very interesting story, which we were able to publish (Busino et al., 2007) despite our initial collaborator becoming our competitor and rushing the submission of their own paper containing data that reproduced ours before we could submit our own paper. Not all collaborators are like Avram…

Where are we going from here? What’s our next step? We aim to apply our biochemical methods to fields heavily dominated by genetics, which struggle with mechanistic studies, similarly to what we have done for the circadian clock field.

### How did you balance the desire to “follow your nose” vs. keeping a focus?

Moving outside to the cell cycle field, I had to study completely different pathways, but learning new things is something I thoroughly enjoy. I was also very fortunate to recruit the right people to the lab, which made it possible for us to take on very different areas of focus. To recruit highly skilled lab members in general is becoming a major challenge today. I was in a lucky position to start my lab at a time when biomedical sciences were exploding (at the time of the doubling of the NIH budget) and many scientists were motivated to go into basic research.

### Over your career, what has been the greatest changes to academia as a whole?

As I mentioned, today a major challenge in science is that the pool of postdocs interested in academic work has tremendously decreased. Americans historically are going into other more financially profitable professions. It is also more difficult to attract talent from outside of the US, countries like Asia and Europe, which populated academic US institutions in the past. At NYUSoM (and elsewhere) we increased the salary of postdocs in an attempt to incentivize people to pursue a postdoctoral experience but I believe that the decreased motivation to go into sciences goes beyond just financial benefits. Science requires great resilience; long work hours, continuously applying for grants, fighting problematic reviewers, and all with no guarantee for success. Still, we do it, because research is not a “job” but rather a “call.” This “bohemian” mentality is, for many reasons, disappearing.

There are other reasons why is difficult to recruit highly skilled talent. These days, there are many good biomedical institutes in Asia and Europe, and people can still do great research staying closer to home. Yet, in my opinion, coming to the US has its advantages and is an experience worth doing. Since I have lived and done research in Europe, I can say that the various European countries are very similar to each other, much more than they like to admit. However, there are substantial differences between Europe and the US. For example, US institutions are often very efficient because they are extremely dynamic, while being less hierarchical and less bureaucratic. Moreover, positive influence from the corporate world has helped US institutions manage and attract money in a more efficient way, which is good for science as extra funds are used to improve infrastructure and facilities. In Europe, research institutes are producing high quality science but sometimes they struggle with bureaucracy for grant applications, animal protocols, etc. A postdoctoral experience in the US promotes independence and can teach the American, dynamic mentality - which is very useful going back to Europe to work. And vice versa, some elements of the European background and mentality can be useful if one wants to remain in the US.

With the crisis of postdocs, I fear that in 10 years many of the research buildings recently built will be left empty as there will be no succession plan for current PIs. Of course, with less PIs, perhaps there will be less competition, more funds/capita, higher salaries, etc.; all things that could increase the quality of science. In fact, there is already a tendency to have smaller but more collaborative labs, although this is still hindered by the old model where the first and last authors in publications get most of the credit, which is opposite to the spirit of collaboration. Collaborations are also spurred by the increasing need of different kinds of expertise. In all cases, though, collaborations need to be spontaneous and never imposed from the top, as sometimes happens because administrators decide so.

### What is your opinion about the climate of publishing today?

Publishing today is a major issue and it is not helping science. The NIH wanted to change things but, ultimately, they merely asked every grantee to make papers available online 1 year after publication. If they wanted to be really bold, they could have done much more than that, by requesting grantees to upload all papers in a dedicated NIH repository in a peer review-free manner. In fact, in my opinion, peer review is overrated and does not impact what is published, just where is published. I believe that peer review does not significantly improve most papers; it definitely increases the time until publication and consumes much more money, yet the core message of the studies remains largely unchanged in most cases. There are also risks associated with peer review. For example, when the authors need the publication to apply for jobs, promotions, or grant applications this generates a conflict of interest, and unreproducible results could result from the pressure put on by reviewers. Moreover, to publish papers in high impact journals, authors may spin the story to make it to appear more interesting. If scientists could publish directly in a repository, time and money would be saved, and potentially this could also lead to more reproducible results. Additionally, all the time and efforts in the review process can be saved and invested into better studies. In other terms, we could save billions of dollars, which include the large amount of scientists’ time spent in peer reviewing papers, by eliminating peer review and the for-profit publishing system. I do realize, though, that this may not be a very popular view…

### How about scientific discussions at conferences? Were they more intense or honest 20–25 years ago compared to now?

When I started with research, the scientific community was smaller, everybody knew each other, and conferences were a sort of work in progress to present new data and hypotheses. There were more discussions, sometimes even animated since when people actual know each other, it was easier to express honest opinions, even if contrasting. Parenthetically, back then, everyone knew if a lab consistently produced work that could not be reproduced, and such a lab would be marginalized unless they corrected their course. Now the community is so large that only a small percentage of people know the rigor of the work in each lab, so, those who cheat have an easier life than they did before.

### How can we ensure quality control?

The only way to assess the quality of a study is to carefully read the paper and for other labs to do studies that confirm the finding. For new recruits and promotions, not only do you have to read their papers, but letters of recommendation play a role. I believe that we should call referees on the phone and listen to their input. It is also beneficial to talk to trainees and colleagues directly. Of course, all of this takes more work and time but the outcome would improve the quality of the scientific community.

### What is exciting to you right now in science?

I am really happy when I find the right people to interact with, both inside and outside my lab. People who are truly interested about noteworthy biological problems. I need an intellectually stimulating environment since, at the end of the day, this is an intellectual job. Yet, intellect and heart cannot be completely separated. This is why we use words as love and passion when talking about this job. As Carlos Castaneda wrote in his book “The Teachings of Don Juan,” this work is worth all the efforts when “*traveling on paths that have heart*.”

### In your office, you display on the wall all the laptops from your career. What is the purpose?

**FIGURE 1:**
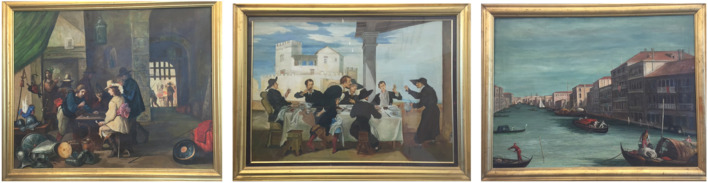
Photo of paintings by Michele’s father, artist Dr. Renato Pagano. Michele jokes that the first two remind him of academic meetings and the last painting reminds him of the view from his office window. Photograph by the authors, published with Dr. Michele Pagano's permission.

**FIGURE 2:**
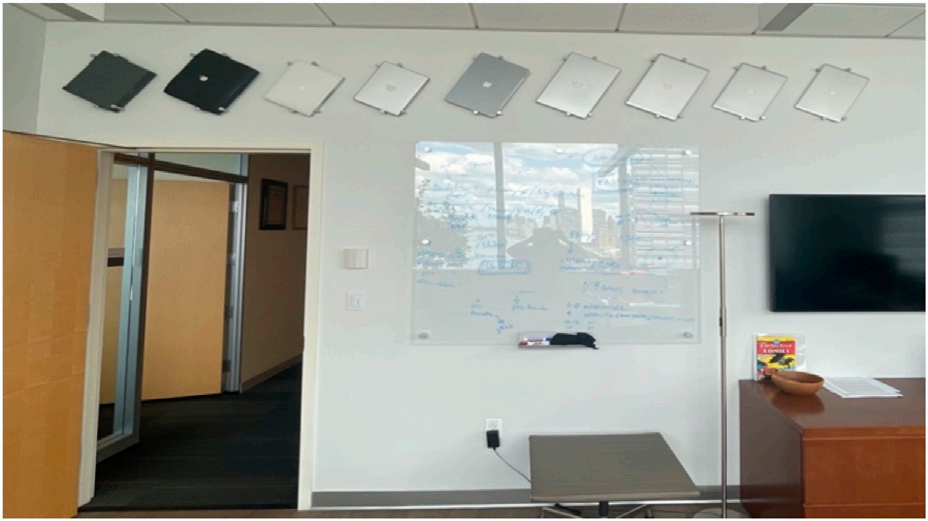
Dr. Michele Pagano’s office wall installation of all his (past) Mac laptops. Photograph by the authors, published with Dr. Michele Pagano's permission.

There will be one more soon! I do not have the talents of my father who can paint (M.P. is pointing to a beautiful oil painting on the wall) (Figure 1). My “Mac installation” (Figure 2) is my way of expressing my own artistic creativity outside of science.

## Conclusion

There are many open questions in the field of ubiquitin research that include elucidating the ubiquitin code, understanding the 3-dimensional structure of various classes of ubiquitin ligases, and determining the physiological functions of ubiquitin ligases. These investigations require the contribution of many laboratories, a lot of time and effort that will help to understand not only basic biology but also the development of diverse diseases.

We thank Michele Pagano for this informative fireside chat. More information about Michele Pagano can be found at: https://med.nyu.edu/faculty/michele-pagano; https://www.hhmi.org/scientists/michele-pagano; https://www.paganolab.org/

## Data Availability

The original contributions presented in the study are included in the article/supplementary material, further inquiries can be directed to the corresponding authors.
